# Representaciones sociales en las personas mayores y su influencia en el autocuidado*

**DOI:** 10.15649/cuidarte.2107

**Published:** 2022-10-16

**Authors:** Ana Victoria Marquez-Terraza

**Affiliations:** 1 . Universidad Nacional de San Luis - CONICET - Universidad de Congreso. Mendoza, Argentina. Email terrazama@gmail.com Universidad de Congreso Universidad de Congreso Mendoza Argentina terrazama@gmail.com

**Keywords:** Cognición, Autocuidado, Salud, Enfermedad, Calidad de Vida, Cognition, Self Care, Health, Disease, Quality of Life, Cognigáo, Autocuidado, Saúde, Doenga, Qualidade de Vida

## Abstract

**Introducción:**

Las representaciones sociales son construcciones que pueden determinar las conductas que las personas mayores llevan a cabo, entre ellas, la conducta de autocuidado. El objetivo de esta investigación fue describir las representaciones emergentes de salud, enfermedad y calidad de vida y su relación con las prácticas de autocuidado de un grupo de personas mayores de la zona este de la provincia de Mendoza.

**Materiales y métodos:**

La metodología utilizada fue cualitativa, utilizando la teoría fundamentada con diseño emergente. La muestra estuvo constituida por 30 personas mayores de 60 años, residentes en la zona este de la provincia de Mendoza, no institucionalizadas, y sin diagnóstico de enfermedad psiquiátrica. Como instrumento se utilizó la entrevista semidirigida.

**Resultados:**

Se encontró que las personas mayores tienen una representación social multidimensional tanto de la salud, como de las causas de la enfermedad y de la calidad de vida, incluyendo en las representaciones elementos del orden físico, psicológico, social y cultural. Esto se encuentra en consonancia con las estrategias variadas de autocuidado que las personas mayores consideran importantes y llevan a cabo.

**Discusión:**

Si bien en la representación social de salud y enfermedad primó la dimensión psicológica, las estrategias de autocuidado que sobresalieron fueron las de tipo físico.

**Conclusiones:**

El comprender las representaciones sociales de la salud, la enfermedad y la calidad de vida de vida de los adultos mayores, puede ser una valiosa herramienta a la hora de diseñar estrategias educativas y de promoción de la salud y del autocuidado para dicha población.

## Abstract (en)

**Introduction:**

Social representations are constructions that can determine the behaviors that older people carry out, including self-care behavior. Objective: This study is aimed to describe emergent representations of health, illness and quality of life and how these relate to self-care practices in a group of older people in the eastern region of the Mendoza province.

**Materials and Methods:**

A qualitative grounded theory study was conducted based on an emerging design with 30 people over 60 years old in the eastern region of the Mendoza province, who were not institutionalized patients and had not been diagnosed with a mental illness before. A non-probability voluntary response sample was used for selection and a semi-structured interview was applied as an instrument.

**Results:**

Older people were found to have a multidimensional social representation of health, illness causes and quality of life, including physical, psychological, social and cultural elements in their representations, which is in line with self-care strategies that older people consider important to carry them out.

**Discussions:**

Although the psychological dimension was given priority in health and illness social representation, the physical self-care strategies were excelled.

**Conclusions:**

Understanding the social representations of health, illness and older people's quality of life can be a valuable tool when designing educational strategies and health and self-care promotion for such population.

## Resumo

**Introdujo:**

As representares sociais sao construyes que podem determinar os comportamentos que os idosos realizam, incluindo o comportamento de autocuidado. O objetivo: desta pesquisa foi descrever as representares emergentes de saúde, doenga e qualidade de vida e sua relagáo com as práticas de autocuidado de um grupo de pessoas idosas na parte oriental da província de Mendoza.

**Materiais e métodos:**

A metodologia utilizada foi qualitativa, utilizando uma teoria fundamentada com um desenho emergente. A populagáo era composta por 30 pessoas com mais de 60 anos de idade, residentes na zona oriental da provincia de Mendoza, náo institucionalizados, e sem diagnóstico de doenga psiquiátrica. A amostra foi selecionada de forma náo-probabilística, utilizando amostragem voluntária dos participantes. Uma entrevista semidirecionada foi usada como um instrumento.

**Resultados:**

Verificou-se que os idosos tém uma representado social multidimensional da saúde, causas da doenga e qualidade de vida, incluindo elementos físicos, psicológicos, sociais e culturais nas suas representares. Isto está de acordo com as várias estratégias de autocuidado que os idosos consideram importantes e levam a cabo.

**Discussao:**

embora a dimensáo psicológica tenha prevalecido na representado social de saúde e doenga, as estratégias de autocuidado que se destacaram foram as físicas.

**Condusdes:**

Compreender as representaos sociais sobre saúde, doenga e qualidade de vida dos idosospode seruma ferramenta valiosa na concepdo de estratégias educacionais e de promodo da saúde e autocuidado para essa populado.

## Introducción

América Latina y el mundo están atravesando un proceso de transición demográfica que ha traído como consecuencia un envejecimiento de la población. Este fenómeno constituye un éxito de la salud pública, en tanto es fruto de un descenso de la fecundidad y un aumento de la expectativa de vida^1^. Sin embargo, en la región este proceso tiene características particulares. Se desarrolla a una velocidad mayor que en los países desarrollados y con un crecimiento acelerado de la demanda de servicios de salud. Esto podría deberse a que la ganancia en supervivencia obedece a una reducción de las enfermedades infecciosas, y no a un avance en la calidad de vida de la población^2^.

Argentina no escapa a esta realidad. Para el año 2020 se estimaba que en el país vivían un poco más de 45 millones de personas. Del total de la población, en ese año, el 11,4% de las personas eran mayores de 65 años. Esta cifra representa un aumento respecto del año 2000, en el que la población de 65 años y más representaba el 9,7% del total. Se espera que esta tendencia permanezca y se acentúe, ya que se calcula que para el año 2050 los mayores de 65 años representarán el 17,3% de la población, y para el 2100 llegarán al 28,3%^1^.

La salud es un sector especialmente afectado por este fenómeno demográfico, ya que se ha producido también una transición epidemiológica. Las enfermedades infecciosas y parasitarias disminuyen, mientras aumentan las crónico-degenerativas^v^. Esto se traduce en mayor riesgo de discapacidad, ya que las enfermedades crónicas son costosas, reducen la calidad de vida de las personas y son una importante causa de discapacidad, amenazando la independencia funcional de las personas mayores^4^.

Frente a este panorama, se vuelve preciso pensar estrategias que permitan abordar esta problemática. Desde los organismos internacionales se recomienda la implementación de políticas que hagan énfasis en la promoción de la salud y la prevención de la enfermedad, y el fomento de estilos de vida saludables, con el objetivo de propiciar la salud a lo largo de toda la vida^3^. Se aboga incluso por un cambio en el paradigma de atención en salud, que hasta el momento ha estado dirigido a tratar enfermedades de duración limitada. Las necesidades sociales y de salud generadas por el envejecimiento suelen ser complejas y de largo plazo, y abarcan diversas áreas del funcionamiento. Por ello, se propone que el sistema de salud debe poder prever y enfrentar de manera proactiva los cambios funcionales y trabajar con las personas mayores para que puedan tener un mejor control de su propia salud^5^.

En este contexto, el autocuidado se vuelve una estrategia clave para la salud de los y las mayores. El mismo puede ser definido como el conjunto de acciones intencionales que una persona lleva a cabo para controlar los factores tanto internos como externos que pueden afectar su salud. Incluye acciones que abarcan el plano físico, psicológico, social, espiritual, medioambiental y económico y que pueden apuntar a la promoción o el mantenimiento de la salud, el tratamiento de la enfermedad o la prevención de complicaciones^6^^,^^7^.

Se ha demostrado que las prácticas de autocuidado son efectivas, en tanto se relacionan con una mayor capacidad funcional y una mejor calidad de vida en las personas mayores^8^^,^^9^. Sin embargo, estas prácticas no siempre son llevadas a cabo, o son realizadas de manera inadecuada. En la puesta en práctica de las acciones de autocuidado, influyen diversos factores. La escolaridad y la edad son los más citados. Las personas a medida que envejecen, realizan menos prácticas de autocuidado. Estas también son menores en las personas con menos años de escolaridad^10^^,^^11^. A su vez, es importante destacar el papel que juegan las creencias y costumbres predominantes en la cultura del sujeto en las prácticas de cuidado de la salud y el sentido que cada sujeto le asigna a las actividades cotidianas^12^.Las costumbres, las tradiciones culturales, las creencias transmitidas en una sociedad que influyen en las prácticas del cuidado de la salud, van a concretizarse en representaciones sociales sobre la salud, la enfermedad, lo saludable, la calidad de vida. Las representaciones sociales, pueden ser definidas como una visión funcional del mundo, compartida por determinado grupo social. Son funcionales en tanto el sujeto puede usarlas para entender e interpretar el mundo que lo rodea y dar sentido a sus conductas. Por ello se considera que las mismas tienen una efectiva incidencia en el comportamiento de las personas, en tanto que tienen la capacidad de orientar dichos comportamientos^13^^-^^15^.

Por ello, se vuelve importante conocer cuáles son las representaciones sociales de salud, enfermedad y calidad de vida de los adultos mayores y constatar si las mismas se relacionan con las prácticas de autocuidado de este grupo.

En Argentina, los estudios sobre la percepción de calidad de vida, salud, y otras variables asociadas, como el bienestar subjetivo, han sido principalmente de índole cuantitativa. Se ha encontrado que la dimensión de satisfacción vital de la calidad de vida, se encuentra relacionada con la edad, el nivel educativo, el trabajo como voluntaria/o, la posibilidad de hacer turismo y el reunirse con amigos^21^. En otros trabajos se ha explorado y constatado la relación entre la calidad de vida (o algunas de sus dimensiones) y el apoyo social percibido^22^^,^^23^. En un estudio de índole cualitativa, en el que se exploraba la percepción de la salud cognitiva de las personas mayores, se encontró que los mismos reportaron problemas relacionados principalmente con la memoria (olvidos) y la atención (distracción y dificultades de concentración)^16^.

En investigaciones de índole cualitativa, que exploraban las representaciones sociales de la salud se ha encontrado un núcleo central fuerte relacionado con el bienestar y la ausencia de enfermedad^17^. Aunque, en otras, se encontró que los adultos mayores tienen una noción multidimensional de la salud, en tanto contemplaban aspectos físicos, psicológicos, sociales y culturales. Lo mismo pasó con el concepto de enfermedad y padecimiento, los cuáles eran vinculados el primero con síntomas físicos y el segundo con malestares físicos, cognitivos y emocionales, además de hacer referencia a sus aspectos culturales^18^. En personas mayores con limitaciones físicas, se ha encontrado que presentan representaciones sociales de la discapacidad ligadas a emociones negativas, pero también a actitudes de afrontamiento y superación^19^.

Las representaciones sociales de la calidad de vida también han sido estudiadas. En un trabajo de investigación llevado a cabo con 100 personas mayores, se encontró que los participantes presentaron seis tipos de significados relacionados con la calidad de vida: Salud y autonomía, familia y redes sociales, trabajo e ingresos, bienestar y estabilidad, sentimientos y afectos, expectativas e intereses personales^20^. En otra investigación, en donde se comparaba la representación social de la calidad de vida en diferentes grupos etarios, se encontró que lo característico en las representaciones de las personas mayores era el pensamiento de que la calidad de vida se obtiene cuando se han realizado una serie de metas, o se han alcanzado ciertos logros en la vida. Es decir, se interpreta la calidad de vida como el resultado de una buena trayectoria de vida^21^.

En un estudio en el que se vinculó las representaciones sociales de la calidad de vida, con las prácticas llevadas a cabo por personas mayores para promoverla, se reportó que la muestra presentaba una representación social de la calidad de vida ligada a los determinantes sociales de la salud y que las prácticas que llevaban a cabo para promoverla eran variadas y acordes al concepto. Esto evidenciaba una adecuada coordinación entre el saber técnico profesional que era transmitido a esta población, con las representaciones que las personas mayores formulaban y las prácticas que llevaban a cabo^22^.

Las representaciones sociales de las personas mayores, no sólo influyen en las prácticas, sino en los resultados de las intervenciones dirigidas a mejorar la salud de los mismos^23^. Conocer el vínculo entre las representaciones sociales que las personas mayores tienen sobre la salud, la enfermedad y la calidad de vida, y las prácticas de autocuidado, permitirá desarrollar estrategias de promoción de la salud adaptadas a la realidad de esta población^19^. Por ello el objetivo de este trabajo es describir las representaciones emergentes de salud, enfermedad y calidad de vida y su relación con las prácticas de autocuidado de un grupo de personas mayores de la zona este de la provincia de Mendoza.

## Materiales y métodos

En la presente investigación se trabajó con una metodología cualitativa, utilizando la teoría fundamentada, la misma implica un proceso de investigación en el que se recopilan y analizan datos de manera sistemática para derivar de ellos una teoría. Esta teoría al ser más próxima a la realidad puede generar conocimientos contextualizados, aumentar la comprensión del fenómeno estudiado y proporcionar una guía significativa para la acción^24^.

La población estuvo constituida por adultos mayores de 60 años, residentes en la zona este de la provincia de Mendoza, no institucionalizados, y sin diagnóstico de enfermedad psiquiátrica. La muestra fue seleccionada de manera no probabilística, utilizando el muestreo de participantes voluntarios. Participaron del estudio 30 sujetos de entre 60 y 82 años, con una edad media de 69,5 (d.e. 6,81). Predominó el sexo femenino, con un total de 22 mujeres y 8 varones. En cuanto a la escolaridad, la mayor parte de la muestra (el 62,1%) sólo tenía escolaridad primaria o inferior, el 3,4% tiene la escuela secundaria incompleta; el 20,6% estudios superiores incompletos, y el 13,8% estudios universitarios completos. La mayor parte de la muestra, el 48,6% está casada/o, el resto está soltero/a (6,7%), divorciado/a (20,7%), y viuda/o (20,7%). Con respecto a la situación laboral, de los 30 adultos mayores entrevistados, 25 eran jubiladas/os, 3 trabajadores activos/as y 2 pensionados/as; en promedio los participantes jubilados/as llevan 7,96 años en esta situación (d.e. 5,93).

Con respecto al estado de salud, del total de participantes, el 79,3 % de la muestra tenía al menos una enfermedad diagnosticada, siendo lo más frecuente que presenten una enfermedad en 12 de los casos, alcanzando un máximo de 7 enfermedades diagnosticada en uno de los casos; mientras que el 20,7 % restante no presenta enfermedades. En cuanto a la variable “ingesta de medicamentos” el 93,1 % de los participantes toman al menos un medicamento, siendo lo más frecuente la ingesta de al menos un medicamento en el 33,3 %, alcanzando un máximo de 10 en uno de los casos; el 6,9 % restante dice no necesitar de la ingesta de los mismos.

Como instrumento se utilizó la entrevista semidirigida. Para la misma se construyó una guía de pautas en base a las variables de estudio, a saber: salud, enfermedad y calidad de vida. Los puntos principales fueron: Percepción de salud, concepto de salud, prácticas ligadas a la salud, cuidado de la salud, relación con el sistema médico, enfermedad, causas de la enfermedad, comportamientos frente a la enfermedad, calidad de vida, dimensiones de la calidad de vida.

Las entrevistas fueron realizadas de manera individual, en el domicilio de los sujetos, aplicadas por profesionales de la salud y estudiantes avanzados de la carrera de Psicología, en el periodo de diciembre 2018-febrero 2019. Antes de comenzar se leyó y explicó a cada participante el consentimiento informado, el cual fue firmado por cada uno. Para garantizar el anonimato, todas las entrevistas fueron transcriptas por una sola persona, quien eliminó los nombres propios de las transcripciones y reemplazó los nombres de los entrevistados por pseudónimos. Los nombres que aparecen a continuación, en los fragmentos textuales tomados del discurso de los sujetos, son pseudónimos, en ningún momento se exponen los nombres reales. Se realizaron un total de 30 entrevistas^25^.

Para los datos cualitativos se utilizó el diseño emergente dentro de la teoría fundamentada. Para comenzar se realizó una transcripción de las entrevistas, las cuales fueron cargadas al programa Atlas. ti versión 7.5.4. Con el programa se llevó a cabo una primera codificación abierta, explorando las significaciones aportadas por los participantes y teniendo como referencia las dimensiones de la calidad de vida aportadas por el instrumento cuantitativo. Después se definió una lista de códigos final y se procedió a la recodificación de las entrevistas. Finalmente se realizó un análisis teórico de las categorías obtenidas.

**Tabla 1:** Datos sociodemográficos

Características	N	Porcentaje
Género		
Femenino	22	73,3
Masculino	8	26,7
Escolaridad		
Primario o inferior	18	62,1
Secundario incompleto	1	3,4
Estudios superiores incompletos	6	20,6
Estudios universitarios completos	4	13,8
Estado civil		
Casado/a	14	48,6
Soltero/a	2	6,7
Divorciado/a	6	20,7
Viudo/a	6	20,7
En concubinato	1	3,4
Cantidad de enfermedades		
1	12	52,2
2	7	30,4
3	2	8,7
4	1	4,3
7	1	4,3
Cantidad de medicamentos		
1	9	33,3
2	7	25,9
3	4	14,8
5	1	3,7
6	4	14,8
7	1	3,7
10	1	3,7

## Resultados

A continuación, se exponen los resultados del análisis del discurso de los sujetos, agrupados en 5 ejes. En los tres primeros ejes se analizan las representaciones sociales que los adultos mayores presentan sobre la salud, la enfermedad y la calidad de vida. En los últimos dos ejes se analizan las prácticas de cuidado de la salud y comportamiento frente a la enfermedad que los participantes refieren. En el Gráfico 1 se puede observar la red de códigos elaborada a partir del procesador Atlas Ti.

**Gráfico 1:**
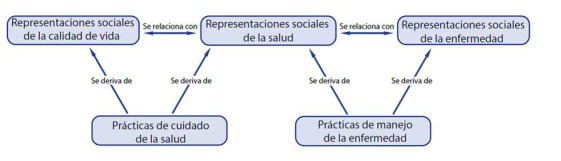
Red de códigos

Representación social dvvve salud: Al preguntarles a los y las participantes por el concepto de salud, se pudo encontrar que las representaciones en torno a este concepto se agrupaban en tres importantes significados. El primero, hace referencia a la salud como una condición indispensable para lo demás. Las personas mayores señalaban que percibían la salud como lo más importante, aquello que posibilita la vida, el trabajo, las relaciones con los demás.

*Es lo más importante que hay. Porque si nosotros no tenemos salud, no se puede trabajar, no se puede vivir, no sé puede hacer muchas cosas que realmente se hacen en la vida. Si uno no tiene salud, no sirve para nada. Alfonso, 70 años.*

Este significado estuvo presente en el discurso de la mayor parte de los sujetos (60%) y debido a su capacidad para nuclear el resto de los enunciados, puede considerarse como el núcleo de la representación de salud.

En segundo lugar, se encuentra gran cantidad de enunciados que relacionan la salud con el bienestar mental, tanto a nivel cognitivo como emocional. Se encuentran enunciados aquí que vinculan la salud con la conservación de las capacidades cognitivas, con la ausencia de penas, con la tranquilidad, o el estar bien con uno mismo. En tercer lugar, se encuentra la concepción física de la salud, aquí se ubican dichos relacionados con la ausencia de dolor, el bienestar físico, la ausencia de enfermedad y la no necesidad de tomar medicamentos. Cabe destacar que se encontraron expresiones que aludían a una concepción social de la salud, pero eran muy reducidas en número para ser consideradas dentro de la representación social.

Representación social de las causas de la enfermedad: En consonancia con el fuerte peso que tenía lo mental en la concepción de salud, en las causas de la enfermedad volvemos a encontrar que la mayoría de los y las participantes presentan representaciones asociadas a lo psicológico. Se repetía con bastante frecuencia la creencia de que las enfermedades tienen origen psicosomático, o que uno se enferma “porque piensa demasiado” o porque “piensa en enfermarse”. También se atribuía el origen de la enfermedad al estrés y a ciertos estados emocionales. Si bien no se pensaba que la enfermedad fuera causada por los factores psicológicos directamente, sí pensaban que estos factores ayudaban a que la enfermedad se manifestara.

*En esta época puede ser el estrés, yo creo mucho en lo psicosomático, que hay enfermedades que se desarrollan por las cuestiones que el organismo tiene, o la mente, o el sistema emocional, y afectan a la salud, entonces ese desequilibrio te lleva a perder la salud. Para mí, no sé, yo no soy médica, lo entiendo así. Fernanda, 75 años.*

En segundo lugar, los participantes mencionaron causas relacionadas con el estilo de vida: el tener hábitos sedentarios, fumar o beber, comer de manera inadecuada, y no cuidarse en general. En tercer lugar, se encuentran causas relacionadas con factores sociales, políticos y económicos. Algunos de los entrevistados concebían la enfermedad como una consecuencia de años de malas condiciones laborales, falta de acceso adecuado al sistema de salud, la pobreza y las preocupaciones que esta condición conlleva.

*Y el desgaste físico del trabajo, mucho trabajo, trabajo en chacras, en viñas por años, tres años trabajé en el contrato, como un hombre, para poder tener esto, que era una sola pieza lo que tenía, no tenía más nada y costar muy mucho para levantarme. Amasar por muchos años, por eso tengo jodida la columna, la cervical, los huesos, los brazos, viste, la cadera. He amasado muy muchos años. Y a puño, nada de amasadora ni nada. Caldear los hornos. Encarnación, 70 años.*

Finalmente, sólo pocas personas identificaron la causa de la enfermedad, en factores biológicos. Específicamente hablaron de herencia genética y virus que invaden el cuerpo y frente a los cuáles no se puede hacer nada, dos de las personas entrevistadas hicieron referencia al COVID-19 como el “virus extraño, chino”. Cabe destacar que estas entrevistas fueron realizadas en el periodo de diciembre de 2019 a marzo de 2020, cuando en Argentina no había gran número de casos confirmados de COVID-19 positivos.

Representación social de calidad de vida: Al preguntarles a los participantes de la investigación por el concepto que ellos tenían de calidad de vida, las respuestas fueron más divergentes. En este caso se pueden ubicar 6 núcleos de significados, sin que uno centralice claramente a los demás. En primer lugar, encontramos la representación de la calidad de vida como la satisfacción de las necesidades básicas, el contar con una casa, los servicios básicos, la comida necesaria, etc. Esta es la representación que más se repite, pero no se articula con el resto de las representaciones emergentes.

*Yo, para mí, que no te falte la comida, que no te falte un par de zapatillas, no sé, por ejemplo, en términos generales. Y poder hacer las cosas que uno quiere. Josefina, 66 años.*

En segundo lugar, encontramos la representación de la calidad de vida como el estar bien con otros y poder disfrutar de la compañía de otras personas. Se menciona aquí el poder llevarse bien con la familia, verlos con determinada frecuencia, compartir actividades con los nietos o con amigos, etc. En tercer lugar, se ubica como una dimensión importante de la calidad de vida el bienestar psicológico, la tranquilidad, la autoestima, la aceptación y la felicidad. En cuarto lugar, encontramos la capacidad de poder vivir en sus propios términos, de manera independiente, siguiendo sus principios y valores.

En quinto lugar, los adultos mayores consideran importante para su calidad de vida, el poder realizar ciertas actividades que provoquen disfrute, diversión y entretenimiento, como los viajes, los paseos, poder darse un gusto. Finalmente, en sexto lugar, los participantes mencionaron la importancia de tener un proyecto o un trabajo para su calidad de vida.

Prácticas de cuidado de la salud: Las prácticas de cuidado de la salud llevadas a cabo por los adultos mayores pueden ser agrupadas en tres diferentes tipos: Prácticas de cuidado físicas, mentales y sociales. Las primeras abarcan una serie de hábitos que apuntan a cuidar el cuerpo en su aspecto biológico. Aquí encontramos conductas como hacer ejercicio, dormir bien, comer bien, no fumar ni beber alcohol, cuidarse al caminar y no levantar cosas pesadas. También incluye la relación con el sistema de salud: los adultos mayores nombran como una conducta de cuidado el ir al médico con cierta frecuencia y tomar los medicamentos que se les recomiendan. Casi la totalidad de los sujetos entrevistados mencionaron uno de estos comportamientos como práctica necesaria para mantener la salud.

*Yo me cuido lo mejor que puedo. Antes iba al gimnasio, pero desde que tuve un problema en el brazo izquierdo, después en la rodilla, y todo eso, no puedo ir al gimnasio. Así que me cuido con las comidas, trato de comer sano, de que todos comamos sano. Por ahí me salgo pero... rara vez. Y bueno, y nada, moviéndome todo el día. Ando, voy y vengo, hago cosas para estar activa, no estar todo el día sentada, porque sé que no puede ser. América, 66 años.*

El segundo tipo de prácticas, las mentales, implican una serie de actividades que el adulto mayor realiza y que colaboran con su salud psicológica, como por ejemplo: ejercitar la mente, mantenerse activo, cuidar el aspecto personal, quererse a sí mismo, tener metas, ir a terapia, divertirse, estar tranquilo/a, mantenerse informado/a. Estas actividades fueron mencionadas como importantes para el cuidado de la salud por la mitad de los participantes.

Finalmente, las prácticas sociales son aquellas que implican un contacto con otras personas. En esta categoría encontramos comportamientos como: no estar aislados/as, hablar con otros y hacer cosas por los demás. Este tipo de comportamientos fue nombrado por algunas personas como importantes para cuidar la propia salud.

Prácticas de manejo de la enfermedad: Frente la aparición de una enfermedad episódica, es decir, no ante una enfermedad crónica, sino ante una enfermedad aguda o una crisis dentro de una enfermedad crónica, las personas adoptan diferentes estrategias. La más utilizada es acudir al médico, y obedecer las recomendaciones del mismo (tomar los medicamentos recomendados y hacerse los estudios solicitados).

Si bien este comportamiento es el más frecuente, no llega a ser el predominante, y es adoptado sólo por algunas personas mayores. Muchos de los participantes dijeron ir al médico sólo en casos extremos, y ante la aparición de algún malestar suelen tratar de mejorarse por sí mismos acudiendo a la automedicación o recurriendo a remedios caseros otros. Dentro de esta última categoría encontramos prácticas como tomar té de diferentes hierbas, usar botellas de agua caliente para aliviar el dolor, o sanar heridas con azúcar.

Finalmente, encontramos un grupo de adultos mayores que toma medidas alternativas, como buscar ayuda en la familia, buscar una distracción, encontrar alivio en la religión o acudir a terapias alternativas.

En el Gráfico 2 se puede observar un resumen de los principales hallazgos agrupados por categorías, en función del análisis expuesto hasta el momento.

**Gráfico 2:**
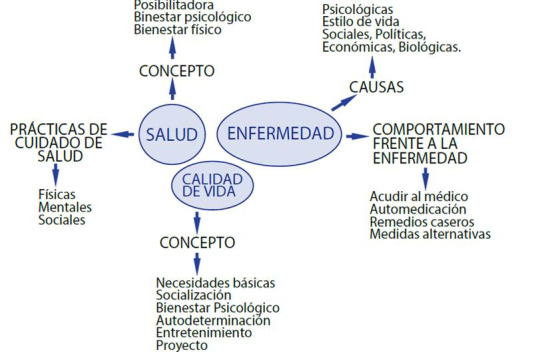
Esquema de los resultados obtenidos

## Discusiones

El objetivo de la presente investigación fue conocer las representaciones que las personas mayores tienen sobre la salud, la enfermedad y la calidad de vida y si las mismas se relacionan con sus prácticas de autocuidado. Se encontró que efectivamente existen una serie de representaciones sobre estos tópicos, que se repiten en los adultos mayores, y pueden ser entendidas como una representación social.

Es necesario recordar que las representaciones sociales son representaciones acerca de un objeto que son compartidas por un grupo social determinado^26^. En este caso, en el grupo de personas mayores, se pudieron constatar diversas representaciones acerca de cada objeto social. Sin embargo, sólo se analizaron aquellas que eran compartidas por varias personas.

Con respecto al objeto social “Salud” se encontró que las personas mayores entrevistadas, tenían una representación social de la salud con un núcleo central bien claro. El núcleo central puede ser identificado tomando en cuenta la frecuencia de aparición de una representación y por su capacidad de generatividad, es decir, por su capacidad para otorgarle sentido y articular al resto de las representaciones que serán identificadas en este caso como periféricas^13^.

La salud está representada por la mayor parte de los adultos mayores como un posibilitador, como una condición necesaria para la vida, para realizar actividades, para trabajar, para relacionarse con otros. Esto coincide con los hallazgos de otros autores, que en investigaciones que exploraban la representación de salud en adultos y adultos mayores, también encontraron que la misma es entendida como una condición asociada al bienestar^17^^,^^27^^,^^28^. Además, se puede constatar la importancia de la salud para el adulto mayor, teniendo en cuenta una investigación que encontró que la palabra salud ocupa el centro de la representación de la vejez para los adultos mayores^28^. Concebir a la salud como una condición posibilitadora concuerda con la propuesta de la Carta de Ottawa, en donde se declaraba que la salud no debía ser entendida como un objetivo, sino como una fuente de riqueza para la vida diaria^30^.

Las representaciones periféricas encontradas se articulan en una dimensión psicológica y una dimensión física de la salud, teniendo mayor peso la primera. También hubo algunas alusiones a la dimensión social de la salud, pero fueron escasas. Se puede ver que existe cierta apropiación por parte de este grupo del concepto de salud propuesto hace más de 40 años por la Organización Mundial de la Salud en la declaración de Alma Ata que la propone como un completo estado de bienestar físico, mental y social^31^ y que aún hoy forma parte de la constitución de esta organización^32^. En otras investigaciones realizadas con adultos y adultos mayores, se ha constatado que la representación de la salud que las personas tienen es eminentemente multidimensional, abarcando aspectos físicos, sociales, culturales, espirituales y psicológicos^18^^,^^33^. En este caso, sólo tuvieron peso las representaciones asociadas a la dimensión psicológica y física, dejando de lado las dimensiones social y cultural.

Sin embargo, al preguntarles por las causas de la enfermedad, las representaciones se volvieron más diversas, y en este caso sí se incluyeron factores comportamentales, socioeconómicos y políticos. Aunque los factores psicológicos volvieron a ser predominantes, y llama la atención que sólo muy pocos participantes mencionaron causas biológicas como el origen de las enfermedades. Esto se contrapone a los hallazgos de otros estudios, en donde la preminencia ha recaído en las concepciones biológicas y físicas de la enfermedad^18^^,^^33^.

A pesar del fuerte acento puesto por los participantes en los aspectos psicológicos de la salud y la enfermedad, las conductas de autocuidado y de acción frente a la enfermedad centraban su accionar en aspectos físicos. Las conductas de autocuidado más frecuentemente mencionadas son aquellas dirigidas a cuidar el cuerpo, tales como hacer deportes, tener una buena alimentación, dormir bien, etc. Sin embargo, es importante destacar que se incluían también, aunque en menor medida, conductas de autocuidado psicológicas y sociales. Esto concuerda con lo encontrado en otras investigaciones donde se reportan prácticas de cuidado que incluían las dimensiones física, mental, social, espiritual y emocional^22^^,^^34^.

Con respecto a las conductas para tratar la enfermedad, se encuentra que si bien hay un porcentaje importante de personas que acuden al médico en primera instancia, un gran porcentaje de personas optan por apelar a recursos definidos por ellos como más accesibles, como por ejemplo la automedicación o la medicina tradicional. Se ha realizado una investigación sobre la representación social de la automedicación en adultos mayores, sin embargo, sólo se concluye que existen tanto connotaciones positivas como negativas asociadas a dicha práctica^35^.

Por último, cabe desatacar que la representación social de calidad de vida, presenta seis dimensiones diferenciadas: satisfacción de las necesidades básicas (agua, vivienda, alimentación), relaciones sociales (estar bien con la familia, hacer cosas por los demás, tener amigos), bienestar psicológico (tranquilidad, autoestima, felicidad), autodeterminación (poder vivir en sus propios términos, de manera independiente y en función de sus propios valores), actividades recreativas (poder realizar actividades como salir de viaje, bailar), y proyectos (tener un trabajo, actividades o proyectos que otorguen un sentido a su vida).La representación social de la calidad de vida encontrada es claramente multidimensional y coincide en parte con los resultados reportados por otros autores quienes dan cuenta de representaciones ligadas a diversas dimensiones en personas mayores^21^^,^^36^. Sin embargo, se diferencia de otra investigación realizada con una muestra de adultos mayores colombianos que en su concepción de la calidad de vida prima la importancia de la vida en comunidad y las ayudas del gobierno^37^. Las personas participantes de este estudio tenían una visión más individualista, ya que incluso al hablar de las relaciones sociales primaba el individuo y las relaciones dentro del círculo familiar: “Llevarme bien con otros”, “estar bien con mi familia”. Esto coincide con el hecho de que, en la representación social de la salud, la dimensión social haya sido escasamente mencionada.

La calidad de vida es un concepto complejo que abarca dimensiones objetivas y subjetivas, que contempla todos los aspectos de la vida humana (emocionales, sociales y físicos, incluyendo la relación con la satisfacción de las aspiraciones de la persona) y que se ve fuertemente influenciado por las características de la sociedad y de la época histórica que se atraviesa^38^^-^^40^. Esta complejidad se ve reflejada en las dimensiones contempladas por los adultos mayores, que incluso mencionaron la importancia de tener un proyecto de vida, o una actividad que de sentido a su vida.

Si bien este estudio tiene sus limitaciones, ya que se utilizaron sólo técnicas cualitativas y el tamaño de la muestra es pequeño, la información puede ser útil para empezar a comprender cuáles son las concepciones de salud y enfermedad que subyacen a las prácticas de autocuidado de los adultos mayores.

## Conclusiones

Al explorar la relación existente entre la representación de salud y origen de la enfermedad y las prácticas de autocuidado de la salud encontramos que existen algunas concordancias y ciertas disonancias. Son concordantes en tanto los adultos mayores tienen una concepción multidimensional de la salud y de las causas de la enfermedad, y a la vez las prácticas de autocuidado que llevan a cabo son multidimensionales.

Sin embargo, existe una discordancia dado que la dimensión psicológica es la predominante en la representación social de salud y de origen de la enfermedad, pero en las prácticas de autocuidado de este grupo predominan las destinadas a cuidar el cuerpo. Es necesario igualmente contemplar qué significado tienen para ellos estas prácticas de índole física ya que, al tener una concepción multidimensional de la salud, los adultos mayores podrían realizar estas prácticas con fines diversos. Por ejemplo, Sofía hace años asistía al gimnasio para ser flexible, pero también porque la ayudaba a superar la muerte de su marido y no tener depresión.

Una mayor comprensión de estas concepciones ayudará a planificar campañas educativas que lleven a mejores y más eficientes prácticas de autocuidado en esta población. La educación para la salud y la apropiación social de los conocimientos por parte de los adultos mayores, sólo tendrá éxito y fomentará el desarrollo de procesos de promoción de la salud y participación social en tanto los saberes y concepciones de este grupo etario sean tenidos en cuenta^41^.

### Aspectos éticos

En la investigación no se llevaron a cabo experimentos con humanos. Las encuestas fueron tomadas habiendo pedido consentimiento de las personas. El consentimiento fue presentado por escrito y de manera verbal, pidiendo retroalimentación a la persona mayor para asegurar una cabal comprensión de lo que se le estaba explicando. Conforme a las normas del Código de Ética del Colegio de Psicólogos de Mendoza, provincia donde se realizó el trabajo de campo, se proveyó información acerca del procedimiento de la entrevista, el manejo de la información de manera confidencial, el uso de la información para publicaciones y la voluntariedad de la participación en la investigación. A los participantes que mostraron acuerdo con esto, se les pidió que dejaran constancia por escrito de su consentimiento.
